# Health behaviors and participation in health promotion activities among hospital staff: which occupational group performs better?

**DOI:** 10.1186/1472-6963-14-474

**Published:** 2014-10-22

**Authors:** Shu-Ti Chiou, Jen-Huai Chiang, Nicole Huang, Li-Yin Chien

**Affiliations:** Health Promotion Administration, Ministry of Health and Welfare, Taipei, Taiwan; Institute of Public Health, National Yang-Ming University, Taipei, Taiwan; Alternative Military Service - Surveillance and Research Division, Health Promotion Administration, Ministry of Health and Welfare, Taipei, Taiwan; Management Office for Health Data, China Medical University Hospital, Taichung, Taiwan; Research Center for Chinese Medicine & Acupuncture, China Medical University, Taichung, Taiwan; Institute of Hospital and Health Care Administration, National Yang-Ming University, Taipei, Taiwan; Institute of Clinical and Community Health Nursing, National Yang-Ming University, 155 Li-Nong Street, Section 2, Bei-Tou, Taipei, 11221 Taiwan

**Keywords:** Health behaviors, Health care staff, Health promotion, Health promoting hospital, Taiwan

## Abstract

**Background:**

Staff health behaviors affect not only their own health but also their provision of health promotion services to their patients. Although different occupational groups work in hospitals, few studies have compared health behaviors among them. The objectives of this study were to examine health behaviors, including physical activity, eating 5 portions of fruits and vegetables per day (5 a day), and stress adaptation, and participation in hospital-based health promotion activities by occupational groups in hospitals.

**Methods:**

This cross-sectional survey was conducted among full-time employees in 100 hospitals across Taiwan. This analysis included 4202 physicians, 31639 nurses, 2315 pharmacists, 8161 other health professionals, and 13079 administrative personnel.

**Results:**

Administrative personnel attended more health promotion lectures and clubs/groups than other health professionals, pharmacists and physicians, and those workers participated more than nurses. Participation in health promotion activities provided by hospitals was associated with better practice of health behaviors. After adjustment for socio-demographics and participation in health promotion activities, physicians, pharmacists, and other health professionals reported more 5 a day than administrative staff. Other health professionals reported more physical activity than administrative staff, and they reported more than physicians. Nurses reported the lowest level of physical activity, 5 a day, and stress adaptation of all occupational groups.

**Conclusions:**

Nurses had worse health behaviors and less participation in health promotion activities than other groups. Workplace health promotion program for health professionals is needed, with special emphasis on nurses. Hospital-based health promotion programs could take the differences of occupational groups into consideration to tailor programs to the needs of different occupational groups.

## Background

The World Health Organization initiated the Health Promoting Hospitals (HPH) Project aiming at reorienting hospitals to integrate health promotion and education, disease prevention and rehabilitation services in curative care [1]. According to the 5 standards for assessment of implementing health promotion in hospitals, emphasis is placed on health promotion among patients, relatives, and staff [1]. In 2012, the Taiwan HPH Network included 76 member hospitals and was one of the largest HPH networks internationally [2]. The Taiwan HPH approach directed Taiwanese hospital leaders’ attention to staff health. Although hospitals increasingly provide health promoting activities for their staff, participation by health care staff and the effects on staff health behaviors have not been studied.

Staff health behaviors affect not only their own health but also their provision of health promotion services to their patients. Zhu et al. [3] reported that normal weight doctors and nurses were more likely than those who were overweight to provide overweight or obese patients with advice and strategies to achieve weight loss. Lobelo et al. [4] asserted that there is compelling evidence that the health of doctors matters and that doctors’ own physical activity habits influence their clinical attitudes and counseling of patients regarding physical activity. General practitioners who smoked themselves were less likely to engage in smoking cessation for their patients [5]. Studies have reported that stress and health risks are high in healthcare workers [6, 7]. Health promotion programs aimed at healthcare workers are suggested to reduce job stress, prevent burnout, improve health, and probably prevent turnover [8, 9].

During the past decades, different occupational groups of health professionals have been increasingly advocating a more health-promoting health services in clinical practices [10–12]. Studies have reported support for reorientation of health services in the incorporation of a greater health promotion [13, 14]. Nonetheless, hospital or acute setting-based health professionals were less likely to engage in health promotion practices than primary health care personnel [13]. If hospital-based health professionals could practice health behaviors themselves, they could not only serve as role models but also influence their attitudes, knowledge, and skills toward health promotion, and hence provision of health promotion services to their patients [15].

Although different occupational groups work in hospitals, few studies have compared health behaviors among them. Such information could be informative in designing hospital-based health promotion programs. The objectives of this study were to examine health behaviors, including physical activity, eating 5 portions of fruits and vegetables per day (5 a day), and stress adaptation, and participation in hospital-based health promotion activities by occupational groups in hospitals. The association between participation in health promoting activities and health behaviors was also examined.

## Methods

### Design and participants

This study was a cross-sectional survey, and included all full-time staff members working in 100 hospitals across Taiwan. We invited all members of the Taiwan HPH project (n = 66 in 2010) to participate in the study. According to the distribution of accredited hospital levels of HPH, we drew a random sample of non-HPH using a 1:1 ratio. Since there were 45 HPH regional hospitals and only 40 non-HPH regional hospitals, all non-HPH regional hospitals were invited, which resulted in a total of 61 non-HPH hospitals. Of the 127 hospitals selected, 100 (78.7%) agreed to participate in this study. Details of the study design are presented elsewhere [16].

We enquired about the number of full-time employees at the study hospitals and distributed questionnaires to each hospital. The staff members were asked to complete and return the anonymous questionnaires in the sealed envelopes provided to a collection site at the hospitals. The study protocol was approved by an Institutional review board at the Bureau of Health Promotion, Department of Health before the inception of the survey (Bureau of Health Promotion investigation number 0990800708).

The survey was conducted from May to July, 2011. A total of 98,817 questionnaires were distributed and 73,391 (74.3%) questionnaires were returned. This analysis included 4202 physicians, 31639 nurses, 2315 pharmacists, 8161 other health professionals, and 13079 administrative personnel.

### Measurements

The data were collected using a structured questionnaire that was developed specifically for this study. The questionnaire was reviewed and modified by six experts and 10 health care workers to ensure its validity.

The study variables included sociodemographic variables (age, sex, educational level, and marital status), work characteristics (accredited hospital level and HPH status), participation in hospital-based health promotion activities, and health behaviors (physical activity, 5 a day, and stress adaptation).

Participation in hospital-based health promotion activities related to physical activity, healthy diet, and stress adaptation was determined by asking “During the past year, did you participate in the indicated activities (including lectures, clubs/groups, and use of equipment)?” Participation in lectures was measured using a 3-point Likert scale ranging from 1 (none), 2 (a couple of times), to 3 (often). Participation in clubs/groups was measured using a 5-point Likert-scale item from 1 (none) to 5 (more than 3 times a week), with a higher score indicating more frequent attendance.

Physical activity and dietary behavior were assessed by enquiring “number of days walking more than 30 minutes or equivalent physical activities during the past week” and “number of days eating 5 portions of fruits and vegetables during the past week”, respectively. Those two questions were assessed using a 5-point Likert scale from 1 (0 day), 2 (1–2 days), 3 (3–4 days), 4 (5–6 days), and 5 (7 days). Stress adaptation was assessed using a self-rated level on a 5-point Likert scale from 1 (very bad), 2 (bad), 3 (still permissible), 4 (good), and 5 (very good).

### Data analysis

Statistical analyses were performed using IBM SPSS Statistics version 18.0 (IBM Corp., Armonk, New York, USA). Categorical variables were characterized by percentage and frequency, while continuous variables were characterized by mean and standard deviation. Differences among occupational groups were examined by χ^2^ statistics and Kruskal Wallis tests. Generalized linear modelling was used to examine the differences in health behaviors by occupational group, with adjustment for sociodemographic characteristics and participation in health-promoting activities. Generalized linear model with one dependent variable can be considered as a multiple regression model, and it allows for the dependent variable to have a normal or non-normal distribution [17].

## Results

Characteristics of the study participants are presented in Table 1. Nurses, pharmacists, and other health professionals appeared to be younger than physicians and administrative personnel (72.4% of nurses, 63.2% of pharmacists, 58.5% of other health professionals, 51.7% of administrators, and 40.9% of physicians were ≤ 35 years old). Roughly 23% of doctors and over 65% of other hospital staff were women (ranging from 68.6% of pharmacists to 98.3% of nurses). Physicians were more likely to be married than those in other groups.

**Table 1:** **Characteristics of study participants; n (%)**

	Physicians	Nurses	Pharmacists	Other health professionals	Administrative personnel	p
	(n = 4202)	(n = 31639)	(n = 2315)	(n = 8161)	(n = 13079)	
Age						<.0001
< 26	34 (0.8%)	5172 (16.7%)	274 (12.2%)	647 (8.1%)	1028 (8.1%)	
26–35	1645 (40.1%)	17271 (55.1%)	1144 (51.0%)	4018 (50.4%)	5522 (43.6%)	
36–45	1215 (29.6%)	6327 (20.4%)	520 (23.2%)	2347 (29.4%)	3662 (28.9%)	
46–55	866 (21.1%)	1871 (6.0%)	253 (11.3%)	834 (10.5%)	1986 (15.7%)	
> 55	339 (8.3%)	341 (1.1%)	52 (2.3%)	132 (1.7%)	480 (3.8%)	
Sex						<.0001
Male	3220 (77.2%)	529 (1.7%)	720 (31.4%)	2056 (25.4%)	2707 (20.8%)	
Female	953 (22.8%)	30984 (98.3%)	1573 (68.6%)	6040 (74.6%)	10297 (79.2%)	
Educational level						<.0001
High school or less	0 (0%)	884 (2.8%)	9 (0.4%)	283 (3.5%)	2341 (18.0%)	
Vocational school	17 (0.4%)	12845 (40.8%)	137 (6.0%)	1264 (15.6%)	3116 (24.0%)	
University	3183 (76.2%)	17032 (54.0%)	1711 (74.6%)	5387 (66.5%)	6229 (48.0%)	
Post-graduate	979 (23.4%)	754 (2.4%)	437 (19.0%)	1170 (14.4%)	1291 (9.9%)	
Marital status						<.0001
Never married	1181 (28.3%)	16989 (53.9%)	1355 (59.1%)	3750 (46.3%)	5544 (42.7%)	
Married	2914 (69.7%)	13751 (43.7%)	897 (39.1%)	4120 (50.9%)	6965 (53.7%)	
Divorced/Widowed	84 (2.0%)	759 (2.0%)	42 (1.8%)	230 (2.8%)	467 (3.6%)	
Accredited hospital level						<.0001
Medical center	1289 (30.7%)	8167 (25.8%)	581 (25.1%)	1956 (24.0%)	3269 (25.0%)	
Regional hospital	2667 (63.5%)	20879 (66.0%)	1523 (65.8%)	5462 (66.9%)	8641 (66.1%)	
District hospital	246 (5.9%)	2593 (8.2%)	211 (9.1%)	743 (9.1%)	1169 (8.9%)	
Certified health promoting hospital						.11
No	1850 (44.0%)	13660 (43.2%)	1026 (44.3%)	3572 (43.8%)	5823 (44.5%)	
Yes	2352 (56.0%)	17979 (56.8%)	1289 (55.7%)	4589 (56.2%)	7256 (55.5%)	

Note: Number in the cell may not match to the total n because of missing data. Missing data were excluded from the analysis listwise for each variable involved.

Physical activity, 5 a day, and stress adaptation significantly differed by occupational group in both Krusal Wallis test and Chi-squared test (Table 2). The practice level was low for all health behaviors with a mean score ranging from 2.10 to 2.99 (between 1–2 days to 3–4 days for physical activity exceeding 30 minutes and 5 a day, and ranging from bad to still permissible for stress adaptation). Physicians reported more days of 30-minute physical activity than administrative staff and other health professionals, followed by pharmacists, who all reported more days of physical activity than nurses. The rate of reporting more than 3 days exceeding 30-minute physical activity was 38.8% for physicians, 35.6% for administrative personnel, 35.4% for other health professionals, 31.8% for pharmacists, and 26.5% for nurses. Physicians reported more days of 5 a day than other health professionals, followed by pharmacists and administrators, who all reported more days than nurses. The rate of reporting more than 5 days of practicing 5 a day was 31.2% for physicians, 27.2% for other health professionals, 25.2% for administrative personnel, 24.7% for pharmacists, and 18.4% for nurses. Nurses had lower stress adaptation than all other groups. The rate of reporting stress adaptation as bad or very bad was 39.9% for nurses and 31% to 32% for other professional groups.

**Table 2:** **Health behaviors among hospital staff; n(%)**

	Physicians	Nurses	Pharmacists	Other health professionals	Administrative personnel	p
	(n = 4202)	(n = 31639)	(n = 2315)	(n = 8161)	(n = 13079)	
Number of days exceeding 30 minutes walking or equivalent physical activity during past week						
Scale; M (SD)	2.40 (1.06)	2.10 (1.10)	2.27 (1.09)	2.35 (1.12)	2.34 (1.11)	<.0001
0 day	785 (18.7%)	10646 (33.6%)	558 (24.1%)	1907 (23.4%)	2969 (22.7%)	<.0001
1-2 days	1787 (42.5%)	12607 (39.8%)	1021 (44.1%)	3357 (41.1%)	5459 (41.7%)	
3-4 days	1008 (24.0%)	4464 (14.1%)	422 (18.2%)	1536 (18.8%)	2574 (19.7%)	
5-6 days	398 (9.5%)	2401 (7.6%)	178 (7.7%)	893 (10.9%)	1347 (10.3%)	
7 days	224 (5.3%)	1521 (4.8%)	136 (5.9%)	468 (5.7%)	730 (5.6%)	
Number of days having 5 portions of fruits and vegetables during past week						
Scale; M (SD)	2.99 (1.10)	2.60 (1.06)	2.82 (1.07)	2.88 (1.09)	2.82 (1.09)	<.0001
0 day	341 (8.1%)	4286 (13.5%)	219 (9.5%)	749 (9.2%)	1335 (10.2%)	<.0001
1-2 days	1112 (26.5%)	11873 (37.5%)	733 (31.7%)	2397 (29.4%)	4017 (30.7%)	
3-4 days	1436 (34.2%)	9662 (30.5%)	790 (34.1%)	2798 (34.3%)	4437 (33.9%)	
5-6 days	887 (21.1%)	3916 (12.4%)	387 (16.7%)	1511 (18.5%)	2237 (17.1%)	
7 days	426 (10.1%)	1902 (6.0%)	186 (8.0%)	706 (8.7%)	1053 (8.1%)	
Perceived adequacy of stress adaptation						
Scale; M (SD)	2.71 (0.79)	2.53 (0.76)	2.66 (0.73)	2.68 (0.73)	2.69 (0.73)	<.0001
Very bad	353 (8.5%)	3691 (11.8%)	188 (8.2%)	601 (7.4%)	969 (7.5%)	<.0001
Bad	938 (22.5%)	8807 (28.1%)	545 (23.8%)	1945 (24.0%)	3063 (23.6%)	
Still permissible	2504 (60.2%)	17579 (56.2%)	1441 (62.8%)	5020 (62.1%)	8067 (62.2%)	
Good	296 (7.1%)	1088 (3.5%)	102 (4.4%)	455 (5.6%)	758 (5.8%)	
Very good	69 (1.7%)	140 (0.4%)	18 (0.8%)	67 (0.8%)	121 (0.9%)	

Note: p value was from Kuskal Wallis test or *X* ^2^ test as appropriate.

Participation in health promotion activities by occupational group is presented in Table 3. The participation level was low for all activities with a mean score ranging from 1.11 to 1.56 (ranging from none to a couple of times). Administrative personnel attended health promotion lectures and participated in clubs more often than other health professionals, and these staff participated more than pharmacists and physicians. Nurses participated the least, except for attending lectures on stress adaptation. Nurses attended lectures on stress adaptation more often than other occupational groups. Physicians used gyms and sports equipment more often than other occupational groups.

**Table 3:** **Hospital staff participation in health promotion activities provided by hospitals; mean (SD)**

	Physicians	Nurses	Pharmacists	Other health professionals	Administrative personnel	p
	(n = 4202)	(n = 31369)	(n = 2315)	(n = 8161)	(n = 13079)	
Attend lectures	1.25(0.48)	1.25(0.46)	1.24(0.47)	1.29(0.49)	1.41(0.55)	<0.0001
Participation in sports-related clubs	1.23(0.62)	1.12(0.46)	1.27(0.72)	1.25(0.68)	1.29(0.74)	<0.0001
Use of gym or sports equipment	1.56(0.99)	1.34(0.73)	1.36(0.77)	1.49(0.90)	1.39(0.83)	<0.0001
Attend lectures	1.18(0.44)	1.17(0.40)	1.14(0.38)	1.20(0.46)	1.24(0.46)	<0.0001
Participation in weight-control groups or activities	1.14(0.39)	1.13(0.35)	1.13(0.36)	1.17(0.41)	1.21(0.44)	<0.0001
Attend lectures	1.24(0.46)	1.48(0.52)	1.24(0.45)	1.30(0.48)	1.38(0.52)	<0.0001
Participation in recreational or service clubs	1.17(0.52)	1.11(0.40)	1.16(0.55)	1.17(0.53)	1.23(0.64)	<0.0001

Note: p value from Krusal Wallis test.

Generalized linear models for the 3 health behaviors are presented in Table 4. Participation in health promotion activities was positively related to the practice of health behaviors. Married staff reported better 5 a day and stress adaptation, but less days of physical activity exceeding 30-minutes than others. Staff with higher educational levels reported better 5 a day and stress adaptation, while they reported fewer days of physical activity exceeding 30-minutes. There were no significant differences in 5 a day and stress adaption between staff of HPH and non-HPH hospitals. Staff of HPH hospitals reported more days exceeding 30-minutes physical activity than non-HPH hospitals. After adjustment for those variables, nurses reported the lowest level of physical activity, healthy diet, and stress adaptation of all occupational groups. Physicians reported less physical activity but more days of 5 a day than administrative staff. Pharmacists reported more days of 5 a day than administrative staff. Other health professionals reported more physical activity and days of 5 a day than administrative staff.

**Table 4:** **Generalized linear models for health behaviors**

	Number of days exceeding 30 minutes physical activity during past week			Number of days having 5 portions of fruits and vegetables during past week			Perceived adequacy of stress adaptation		
	Estimate (S.E.)	95% C.I.	P-value	Estimate (S.E.)	95% C.I.	P-value	Estimate (S.E.)	95% C.I.	P-value
Professional background									
Physicians	-0.07 (0.02)	-0.11, -0.03	.002	0.11 (0.02)	0.07, 0.15	<.0001	-0.01 (0.02)	-0.04, 0.02	.34
Nurses	-0.10 (0.01)	-0.12, -0.07	<.0001	-0.12 (0.01)	-0.15, -0.10	<.0001	-0.13 (0.01)	-0.15, -0.12	<.0001
Pharmacists	-0.04 (0.03)	-0.09, 0.01	.12	0.07 (0.02)	0.02, 0.12	.006	-0.01 (0.02)	-0.04, 0.03	.60
Other health professionals	0.06 (0.02)	0.03, 0.09	<.0001	0.09 (0.02)	0.06, 0.12	<.0001	-0.02 (0.01)	-0.01, 0.04	.16
Age									
26-35	-0.14 (0.01)	-0.17, -0.11	<.0001	0.11 (0.01)	0.09, 0.14	<.0001	0.01 (0.01)	-0.02, 0.03	.60
36-45	-0.09 (0.02)	-0.13, -0.06	<.0001	0.33 (0.02)	0.30, 0.37	<.0001	0.04 (0.01)	0.02, 0.07	.001
46-55	0.14 (0.02)	0.10, 0.19	<.0001	0.48 (0.02)	0.43, 0.52	<.0001	0.15 (0.02)	0.12, 0.18	<.0001
>55	0.34 (0.03)	0.27, 0.41	<.0001	0.60 (0.03)	0.54, 0.67	<.0001	0.23 (0.02)	0.18, 0.28	<.0001
Sex									
Female	-0.35 (0.02)	-0.38, -0.32	<.0001	0.08 (0.01)	0.05, 0.11	<.0001	-0.03 (0.01)	-0.05, -0.01	.01
Educational level									
Vocational school	-0.16 (0.02)	-0.21, -0.12	<.0001	.00 (0.02)	-0.04, 0.04	.97	-0.03 (0.02)	-0.06, -0.002	.04
University	-0.22 (0.02)	-0.26, -0.18	<.0001	0.03 (0.02)	-0.007, 0.08	.11	0.002 (0.01)	-0.03, 0.03	.87
Graduate school	-0.24 (0.03)	-0.29, -0.18	<.0001	0.14 (0.03)	0.10, 0.19	<.0001	0.07 (0.02)	0.03, 0.10	<.0001
Marital status									
Married	-0.09 (0.01)	-0.11, -0.07	<.0001	0.18 (0.01)	0.16, 0.21	<.0001	0.02 (0.008)	0.003, 0.03	.02
Divorce/Widowed	0.01 (0.03)	-0.04, 0.07	.63	0.02 (0.03)	-0.04, 0.08	.47	-0.01 (0.02)	-0.05, 0.03	.58
Accredited hospital level									
Regional hospital	0.00 (0.01)	-0.02, 0.02	.98	0.03 (0.02)	-0.001, 0.07	.06	0.00 (0.01)	-0.01, 0.02	.70
District hospital	0.02 (0.02)	-0.02, 0.05	.86	0.03 (0.01)	0.01, 0.05	.002	-0.01 (0.01)	-0.03, 0.02	.47
Certified health promoting hospital									
Yes	0.02 (0.01)	0.00, 0.04	.04	0.01 (0.01)	-0.01, 0.03	.24	-0.006 (0.01)	-0.02, 0.01	.31
Participation in health promotion activities provided by hospitals									
Attend lectures	0.12 (0.01)	0.10, 0.14	<.0001	0.12 (0.01)	0.10, 0.14	<.0001	0.14 (0.01)	0.12, 0.15	<.0001
Participation in clubs/groups	0.08 (0.01)	0.06, 0.10	<.0001	0.07 (0.01)	0.05, 0.09	<.0001	0.06 (0.01)	0.05, 0.07	<.0001

Note: The reference groups were administrative personnel, age <26 years, male, educational level of high school or less, never married, medical center, and non- health promoting hospitals.

## Discussion

We found that nurses had the worst health behaviors and lowest participation in health promotion activities of all occupational groups working in hospitals. A previous study reported that physicians and nurses felt the same level of job stress, and they experienced higher levels of stress than administrative workers in hospitals [6]. Although they had the same stress level, nurses had less support from supervisors and coworkers than physicians. As a result, nurses showed higher stress responses than physicians and administrative workers in hospitals [6]. Our results concurred with a previous study showing that nurses reported worse stress adaptation than other occupational groups [6]. In addition, we found that nurses also reported the lowest level of physical activity and 5 a day. Previous researchers asserted that work-related stress was negatively associated with health behaviors [18, 19]. Thus poor stress adaption and high stress levels may be contributory factors for the low physical activity and 5 a day among nurses. Since the nursing shortage is a serious issue in Taiwan as well as in many other countries [20, 21], hospital administrators should be aware of the local understanding of occupational stressors and productively engage nurses in identifying initiatives to reduce occupational stress, improve stress management, and promote health, which could help prevent burnout and decrease turnover [9, 22, 23]. The finding that nurses attended lectures on stress adaptation more often than other occupational groups suggests that hospital administrators and nurses were aware of the problem of job stress among nurses. Nonetheless, effective strategies other than lectures should be developed.

Mobilizing health promotion among nurses could have great impact on population health since nurses are the largest health professional workforce [24]. Previous studies reported a lack of understanding of the nature and practice of health promotion among nurses in hospital settings [25, 26]. Many nurses defined and practiced health promotion in the narrower terms of health education, i.e., isolated information-giving and disease prevention activities alone [27, 28]. We felt that nurses could be empowered to reflect on their own practice of health behaviors as an individual and a professional group and incorporate systems thinking and socio-ecological models of health promotion into their clinical nursing practices [24]. Further studies are needed to develop and evaluate innovative programs in this aspect.

Administrative workers attended health promotion lectures and participated in clubs/groups more often than other groups and their level of physical activity appeared to be better, but their consumption of 5 a day appeared to be worse than for physicians and pharmacists. Physicians used gyms and sports equipment more often than other occupational groups, but they participated in lectures and clubs/groups less often. Those results reflected differences among occupational groups in hospitals. Hospital-based health promotion programs could take differences in occupational groups into consideration to tailor programs to the needs of different occupational groups.

In general, hospital workers practiced physical activity, 5 a day, and stress adaption at a level that is less than desirable. Participation in health promotion activities was related to better health behaviors among hospital staff, but their level of participation was generally low. More effort is needed to motivate staff to participate in hospital-based health promotion activities, especially nurses, pharmacists, and physicians. Workplace health promotion programs for health professionals are needed, with special emphasis on nurses.

It was noted that 0.4% of physicians reported an educational level of vocational schools; 0.4% of pharmacists, 2.89% of nurses, and 3.5% of other health professionals reported an educational level of high school; 18.0% of administrative personnel reported an educational level of high school or less. Majority of the physicians in Taiwan graduated with a medical doctor degree (University). However, very few physicians could graduate long-time ago. Back in that time, people with a vocational school degree from medical vocational school plus years of medical practices could take qualification examinations and became doctors. Some high schools in Taiwan offered vocational training. Health professionals besides doctors could hold a high school degree if they received vocational training as a health professional in high schools. Administrative personnel could receive an educational level of high school or less. The report of educational level among our participants was within possible ranges, though the distribution would be different from other country context.

This study was limited by the use of a cross-sectional design and causal relationships could not be established. The data were self-reported and we had no way to validate the answers. The study variables were measured by questions developed specifically for this study, rather than standard instruments, which could increase the likelihood of misclassification and limits comparisons with other studies. The study results could be bound to the Taiwanese hospital context and may not be applicable to other systems across the world.

## Conclusions

Of the occupational groups working in hospitals, nurses had the lowest levels of physical activity, 5 a day, and stress adaptation. Nurses attended lectures about stress more often than the other occupational groups, but their stress adaption was poorer. Administrative personnel attended more lectures and clubs/groups related to physical activity and healthy diet than other occupational groups. Hospitals workers practiced health behaviors and participated in health promoting activities at a low level. Participation in health promotion activities provided by hospitals was positively associated with the practice of health behaviors. Workplace health promotion program for health professionals is needed, with special emphasis on nurses. Hospital-based health promotion programs could take the differences of occupational groups into consideration to tailor programs to their needs.
